# Candidate Biomarkers in Children with Autism Spectrum Disorder: A Review of MRI Studies

**DOI:** 10.1007/s12264-017-0118-1

**Published:** 2017-03-10

**Authors:** Dongyun Li, Hans-Otto Karnath, Xiu Xu

**Affiliations:** 1 Division of Child Health Care, Children’s Hospital of Fudan University, Shanghai, 201102 China; 2 Division of Neuropsychology, Center of Neurology, Hertie Institute of Clinical Brain Research, University of Tübingen, 72076 Tübingen, Germany; 3 Department of Psychology, University of South Carolina, Columbia, SC 29208 USA

**Keywords:** Autism spectrum disorder, Biomarker, Neuroimaging, Structural MRI, Diffusion tensor imaging, Resting-state functional MRI, Magnetic resonance spectroscopy, Children, Human

## Abstract

Searching for effective biomarkers is one of the most challenging tasks in the research field of Autism Spectrum Disorder (ASD). Magnetic resonance imaging (MRI) provides a non-invasive and powerful tool for investigating changes in the structure, function, maturation, connectivity, and metabolism of the brain of children with ASD. Here, we review the more recent MRI studies in young children with ASD, aiming to provide candidate biomarkers for the diagnosis of childhood ASD. The review covers structural imaging methods, diffusion tensor imaging, resting-state functional MRI, and magnetic resonance spectroscopy. Future advances in neuroimaging techniques, as well as cross-disciplinary studies and large-scale collaborations will be needed for an integrated approach linking neuroimaging, genetics, and phenotypic data to allow the discovery of new, effective biomarkers.

## Introduction

Autism spectrum disorder (ASD) is a neurodevelopmental disorder that has a strong genetic basis, a heterogeneous etiology, and various clinical presentations. The core symptoms of ASD include: (1) deficits in social communication and social interaction, and (2) restricted, repetitive patterns of behavior, interests, or activities [1]. The prevalence of ASD has dramatically increased recently from 4 in 10000 to 1 in 68 children [2, 3]. Along with the soaring incidence of ASD, significant financial and emotional costs to the individuals and their families, as well as the numerous pressures on our medical, social, and political lives have been engendered [4]. Hence, determining the causes and making an accurate diagnosis as well as early and effective intervention seem crucial for society [5].

So far, the diagnosis of ASD and the selection criteria for clinical trials have been guided by the Diagnostic and Statistical Manual of Mental Disorders or behavioral diagnostic scales. The apparent disadvantage of symptom-based criteria is that a similar symptom or phenotype may arise from diverse sets of biochemical processes, especially for disorders with numerous genetic or environmental factors like ASD [6]. In the past decade, searching for genetic biomarkers has been one of the hottest spots in ASD research. Numerous related genes have been reported including *NRXN1*, *SHANK3*, *SHANK2*, *MECP2*, *SNC2A*, *CHD8*, *DYRKIA*, *POG2*, *GRIN2B*, *KATNAL2*, *NLGN3*, *NLGN4*, *CNTN4*, *CDH10*, *CDH9*, and *SEMA5A* [7–17]. Unfortunately, the genetic underpinnings of ASD are neither simple nor consistent, considering that only 10%–38% of ASD cases have been reported with known genetic deficits [18–20]. Because of this heterogeneity and complexity, interest and efforts into searching for more biomarkers and quantifiable parameters are increasing in order to facilitate early and reliable diagnosis, as well as to subgroup patients sharing common pathophysiological underpinnings.

Magnetic resonance imaging (MRI), a non-invasive examination tool, has been widely applied to ASD populations to delineate the developmental trajectory of the brain. Major advances in structural and functional MRI techniques in the past decades have greatly enriched our understanding of neuropathological differences in ASD [19, 21, 22]. Generally, structural MRI has revealed ASD to be a disorder with general and regional brain enlargement, especially in the frontotemporal cortices, while functional MRI studies have highlighted diminished connectivity, especially between frontal-posterior regions [19, 21–25].

Here, we review recent MRI studies (since 2000) in young children with ASD, aiming to provide effective biomarkers for the diagnosis of childhood ASD. We focus on studies using structural imaging methods, structural connectivity analyses, diffusion tensor imaging (DTI), neurochemical or metabolic quantification methods, and magnetic resonance spectroscopy (MRS), as well as functional connectivity analyses with resting-state functional MRI (rs-fMRI). We do not consider task-based functional methods, since it is almost impossible to keep a young child awake and still during a functional scan. We place the emphasis on young ASD children because in adolescents and adults the altered brain structures and activities may merely reflect the social deprivation experience elicited by reduced social attention during childhood. Therefore, it is impossible to tell whether observed functional or structural differences are the cause or the result of ASD neuropathology. Another reason is based on brain plasticity. There is growing evidence that the first 3 years of life is a particularly critical developmental period for children with ASD [26, 27]. Thus, the earlier the abnormal neurodevelopmental trajectory (even in infants and toddlers) is identified, the better guided intervention strategies for ASD children can be achieved.

## Structural MRI

Structural MRI analysis for neurodevelopmental disorders began to emerge in the 1990s when it focused on the neuroanatomical aspects of brain development. It has been used to measure the total brain volume and volumes of specific structures. Earlier studies used manual delineation for the gray (GM) and white matter (WM) to calculate the volumes of specific regions of interest (ROIs). With technical developments, it is now possible to use program codes to measure the volumes automatically, allowing large data sets to be processed more efficiently [28]. Based on the different analytic methods for structural data, structural MRI studies can be classified into voxel-based morphometry (VBM) and surface-based morphometry (SBM) [29]. VBM targets tissue density and usually focuses on relative GM concentration or volume, or regional volume differences of a certain tissue. SBM addresses topological features, like surface curvature and degree of folding [29, 30]. Notably, brain volume and surface curvature have been hypothesized to have dissociable developmental trajectories with, putatively, different genetic and neurodevelopmental bases [22, 31, 32]. Table 1 summarizes the findings of structural MRI studies in children.

**Table 1:** Structural MRI studies of children with ASD.

Reference	Age range	Brain regions	Methods	Findings in ASD group
Elia *et al.* (2000) [57]	5–17 years	Corpus callosum; midbrain; cerebellar vermis	Area measurements; T-test; regression	No abnormalities in the total vermis, vermis lobules VI-VII, pons, and midbrain
Carper *et al.* (2002) [35]	2–4 years	WM and GM volumes	ROI; SPSS	↑ WMV in frontal and parietal lobes
				↑ GMV in frontal and temporal lobes
Sparks *et al.* (2002) [36]	3–5 years	Cerebrum; cerebellum; amygdala; hippocampus	ROI; SPSS; ANCOVA	↑ TBV and amygdala, cerebellar, and hippocampus volume
Herbert *et al.* (2003) [51]	7–11 years	Cerebrum; cerebellum	ROI; semi-automated segmentation; SPSS; GLM	↑ TBV and total cerebellar volume
Akshoomoff *et al.* (2004) [37]	4–6 years	Cerebrum; cerebellum; cerebellar vermis; TBV	ROI; ANOVA; segmentation	Low-functioning autism: ↑ TBV and cerebral volume; ASD: ↑ TBV, cerebral and cerebellar GMV and WMV, anterior and posterior cerebellar vermis area
McAlonan *et al.* (2005) [56]	10–12 years	GM; WM regional density	VBM; BAMM; SPSS; GLM; MANCOVA	↓ GM density in frontal and parietal areas; ↓ WM density in cerebellum and left internal capsule and fornices
Hazlett *et al.* (2006) [38]	1.5–3 years	Cerebrum; cerebellum	ROI; NLMM; segmentation	↑ GMV and WMV in cerebrum
Hardan *et al.* (2006) [42]	8–13 years	Cortical thickness	SBM; Freesurfer	Total cerebral sulcal and gyral thickness; no significant difference in frontal and occipital areas
Munson *et al.* (2006) [62]	3–4 years	Cerebrum; amygdala; hippocampus	Area measurements; linear modal	↑ Right amygdala volume
Schumann *et al.* (2010) [39]	1.5–5 years	Cerebrum	ROI; SPSS; ANCOVA; segmentation	↑ GMV and WMV in cerebrum; notably in frontal, temporal, and cingulate cortices
Jiao *et al.* (2010) [44]	7–11 years	Cortex	SBM; T-test; Freesurfer	↑ Thickness in left caudal anterior cingulate cortex and left frontal pole; ↓ thickness in right entorhinal, right lateral orbitofrontal, left lateral orbitofrontal, right medial orbitofrontal, left medial orbitofrontal cortex, and right pars triangularis
Hazlett *et al.* (2011) [34]	6–7 months	Cerebrum; cerebellum	ROI; GLM; automatic segmentation; ANOVA	No significant difference in TBV, cerebral cortex, cerebellum, or lateral ventricle volumes
Shen *et al.* (2013) [40]	(Longitudinal) 6–9 months; 13–14 months; 19–21 months	Cerebrum	ROI; LMM; manual segmentation	↑ CSF over frontal lobe at 6–9 mos; ↑ total cerebral volumes at 12–15 mos
Nordahl *et al.* (2012) [63]	2–4 years	Amygdala	ROI; ANCOVA	↑ Amygdala volume at both time points
Dierker *et al.* (2015) [45]	9–12 years	Cortex	SBM; ANOVA; freesurfer	Bilateral differences in sulcal depth in the anterior-insula, frontal-operculum, and temporal-parietal junction
Frazier *et al.* (2009) [64]	7–12 years	Corpus callosum	ROI; area measurement	↓ Volume of corpus callosum
Barnea-Goraly *et al.* (2014) [79]	(Longitudinal) 8–12 years; 10–14 years	Amygdala; hippocampus	Area measurement; rm-ANOVA	No difference in hippocampus of both hemispheres

WM, white matter; GM, grey matter; TBV, total brain volume; ROI, region of interest; SBM, surface-based morphometry; VBM, voxel-based morphometry; GLM, general linear models; ANOVA, analysis of variance; ANCOVA, analysis of covariance; rm-ANOVA, repeated measures ANOVA; LMM, linear mixed models; NLMM, non-linear mixed models; SPM, Freesurfer, ad SPSS are data analysis packages.

### Cortex

The most consistent observation regarding structural cortical changes in young ASD children *versus* controls is the increased total volume both in the cortical GM and WM [22, 30, 33, 34]. Increased GM volume (GMV) and WM volume (WMV) have been found in the frontal and temporal lobes and are less pronounced in the parietal and occipital lobes. For example, Carper *et al.* (2002) revealed increased frontal and temporal GMV and frontal and parietal WMV at young ages (2–4 years old) [35]. Sparks *et al.* (2002) likewise observed increased cerebral volumes in autistic children aged 4–6 years [36]. Akshoomoff *et al.* (2004) reported increased total brain volume (TBV), as well as significantly increased WMV and GMV in children of 4–6 years with low-functioning autism compared with high-functioning autism (HFA) [37]. Hazlett *et al.* (2006) investigated 51 younger autistic children and 25 typically-developing controls (aged 18–35 months). They reported increased cerebral GMV and WMV in the brains of the autistic group [38]. In a group of 1.5–5 year-old autistic children, Schumann *et al.* (2010) found no changes in the occipital lobe but enlarged cerebral GMV in the frontal, temporal, and parietal lobes, as well as in the cingulate gyrus (located in the limbic lobe) [39]. Hazlett *et al.* (2011) compared the TBV of 6-month-old high-risk infants with autistic siblings with low-risk infants without autistic family members. They found no significant difference in the cerebrum or lateral ventricle volumes, consistent with the hypothesis that the brain enlargement might be a postnatal event, occurring at ~12 months of age [34]. However, Shen *et al.* (2013) identified excessive cerebrospinal fluid (CSF) over the frontal lobes at 6–9 months of age but no such difference at 12–15 and 18–24 months [40]. Recently, Gori *et al.* (2015) extracted features from GM, WM, and CSF measurements to classify autistic and control brains in 4-year-old males. They found that only GM features in different sub-regions showed up to 80% classification performance [41].

SBM studies have only been performed with older autistic children. For instance, Hardan *et al.* (2006) applied atlas-based SBM to children aged 10 years. They found no changes in the frontal or occipital lobes but increased total cerebral sulcal and gyral thicknesses in the temporal and parietal lobes [42]. Two years later, Hardan *et al.* (2009) performed follow-up scans on the same group of autistic children, and revealed decreased cortical thickness in the frontal, temporal, and occipital lobes compared to controls [43]. Moreover, another SBM study by Jiao *et al.* (2010) on 9-year-old autistic children showed decreased thickness in the right entorhinal, right lateral orbitofrontal, left lateral orbitofrontal, right medial orbitofrontal, left medial orbitofrontal cortex, and right pars triangularis, as well as increased thickness in the left caudal anterior cingulate cortex and left frontal pole [44]. More recently, Dierker *et al.* (2015) found bilateral differences in sulcal depth in the anterior-insula, frontal-operculum, and temporal-parietal junctions [45].

According to the above studies, the main differences in the cerebrum of young autistic children are the enlarged total volume of both GM and WM. The most prominent differences were found in the frontal and temporal lobes as well as precentral regions. The growing cerebral volume in early childhood indicates simultaneously decreasing GM and WM density [40]. Interestingly, the changes seem to begin in the autistic cerebrum at the age of ~12 months, i.e., the cerebral enlargement does not occur earlier than 12 months of age [34, 46].

### Cerebellum and Subcortical Areas

#### Cerebellum and Vermis

Cerebellar dysfunction may play a crucial role in the etiology of ASD [47, 48]. The cerebellum is considered to be one of the most consistent sites of abnormality in autism [49, 50]. Most morphometric studies of ASD children have reported increased total cerebellar volume [36, 51, 52] and decreased GMV in some subregions, e.g., crus I and lobules VIII and IX [53–55]. However, the results on WMV have been inconsistent. For instance, Courchesne *et al.* (2001) found a larger cerebellar WMV in 2–3 year-old autistic children [33], while McAlonan *et al.* (2005) reported decreased WM density in the cerebellum [56]. Regarding the vermis, the results are also contradictory. In older children, Elia *et al.* (2000) measured the sizes of the cerebellar areas in 22 autistic children and reported no abnormalities in the total vermis, vermis lobules VI–VII, pons, and midbrain [57]. In contrast, Akshoomoff *et al.* (2004) revealed larger cerebellar WMV and GMV and increased areas of the anterior and posterior cerebellar vermis in 6-year-old autistic children [37].

#### Amygdala

The amygdala has been the focus of several studies in children with ASD, given its important role in socio-emotional processing [58–61]. Sparks *et al.* (2002) investigated 29 autistic children and typically-developing controls with an average age of 3.9 years and showed bilateral enlargement of the amygdala that was proportional to the overall increase in total cerebral volume [36]. Munson *et al.* (2006) argued that the enlargement was only found in the right amygdala at the age of 3–4 years [62]. Nordahl *et al.* (2012) scanned 85 autistic children aged 37 months on average, and re-scanned 45 members of the same group one year later. They found an increased amygdala volume in both cases, confirming that the enlargement starts at ~3 years [63].

#### Corpus Callosum

A decreased corpus callosum (CC) size in the autistic population has been consistently reported, including reductions localized in the mid-sagittal area, anterior CC, body, and posterior splenium [2, 22, 43, 64–69]. A reduced CC has been associated with reduced integration of information and slower processing [2, 68, 69]. However, to our knowledge, CC changes in the early childhood period have not yet been studied. A report by Frazier *et al.* (2012) investigated 19 autistic children with a mean age of 10.6 years and found consistent reductions in the total CC volume [65].

#### Basal Ganglia

The basal ganglia play an important role in cognition and motor control *via* participation in frontostriatal, thalamocortical, and limbic circuits [70–73]. In neuroimaging studies of ASD, the basal ganglia have been studied regarding their association with repetitive behaviors [74, 75]. The most consistent finding is an increased caudate volume [76, 77]. Unfortunately, most of the studies were conducted in adults or late adolescence but not in childhood.

#### Hippocampus

Sparks *et al.* (2002) found enlarged hippocampi in autistic children at the ages of 3–4 years [36]. Similarly, in 7.5–12.5 year-olds, Schumann *et al.* (2004) also revealed enlarged hippocampi, especially in an HFA group [78], while in a 10 year-old group, Barnea-Goraly *et al.* (2014) observed an enlargement only in the right hippocampus [79]. Interestingly, in an older group, Barnea-Goraly *et al.* (2014) reported no difference in the hippocampus of both hemispheres. These findings indicate that the hippocampus in ASD has an unbalanced growth pattern during early brain development [79].

#### Conclusions

The most consistent structural MRI finding is the increased growth of total cortical volume in early ASD children. A similar trend has also been demonstrated in some subcortical brain regions (e.g., amygdala and hippocampus) and the cerebellum. Interestingly, studies comparing ASD adolescents or adults with controls did not find such differences and have even reported decreased TBV in ASD patients [35, 80]. Thus, it seems as if brain development during early childhood in ASD is more voluminous than in a typically-developing brain, especially in the frontal and temporal lobes, followed by a possibly reduced volumetric capacity of the brain after adolescence [81, 82].

### Diffusion Tensor Imaging (DTI)

Molecular studies have demonstrated dysmaturation of the WM characterized by microstructural changes or disorganization in the brains of autistic populations [83, 84]. It has been reported that synaptogenesis is altered in children with ASD, affecting myelination, and thus compromising WM integrity [85]. DTI, as a non-invasive tool, has been applied in the last decade to developing brains to study both the local connectivity and WM tracts as well as fasciculi that connect regions and lobes [2, 86–90]. Metrics such as fractional anisotropy (FA), and mean diffusivity (MD) have been used to measure the directionality and the amount of diffusion, respectively, in a particular ROI or at the level of individual voxels. The most commonly-used DTI methods are tractography, voxel-wise analysis, and tract-based spatial statistics (TBSS). Table 2 summarizes the findings of DTI studies in children.

**Table 2:** Diffusion tensor imaging studies of ASD children.

Reference	Age range	Brain regions	Methods	Findings in ASD group
Sundaram *et al.* (2008) [91]	2–7 years	Association fibers in frontal lobes	DT; ROI; MANCOVA; DTIstudio	↑ MD in short and long-range fibers; ↓ FA in short-range fibers
Hong *et al.* (2011) [92]	7–11 years	Corpus callosum (CC)	DT; ROI; WM density and volume; FSL; SPSS	↓ WM density in the anterior third of the CC; ↑ MD in the anterior third of the CC
Nagae *et al.* (2012) [93]	7–18 years (+/-language impairment)	Superior longitudinal fasciculus (SLF); corticospinal tract (CST)	DT; GLM; SPSS; DTIstudio	↑ MD in CST (ASD without language impairment); ↑ MD in left SLF (ASD with language impairment)
Wolff *et al.* (2012) [94]	(Longitudinal) 6–7 months; 12–13 months; 23–25 months	Global main tracts	DT; ROI; DTIprep; SAS	↑ FA in CC body, left fornix, inferior longitudinal fasciculus, uncinate fasciculus (6 mo); no difference in FA (12 mo); ↓ FA in left anterior internal capsule and anterior thalamic radiation (24 mo)
Nair *et al.* (2013) [95]	9–17 years	Cortex; thalamus	PT; ROI; FSL; T-test	↑ MD in thalamo-cortical connectivity; negative correlation between fronto-thalamic FA and ADOS score
Joseph *et al.* (2014) [96]	4–6 years	Arcuate fasciculus	PT; ANOVA; FSL	↓ RD of arcuate fasciculus
Cheung *et al.* (2009) [97]	6–14 years	Global white matter	VBM; SPM; GLM; SPSS	↓ FA in bilateral prefrontal and temporal regions; ↑ FA in SLF and left occipital lobe
Ke *et al.* (2009) [98]	6–11 years	Regional frontal and temporal gyri	VBM; SPM	↓ WM density in right frontal and left parietal lobe; ↓ FA in frontal left temporal lobe
Barnea-Goraly *et al.* (2010) [99]	9–14 years	Regional white matter	VBM; FSL	↓ FA in frontal, temporal and parietal lobes
Poustka *et al.* (2012) [100]	8–12 years	Fornix; SLF; uncinate fasciculus; CC	VBM; DT; SPM	↓ FA in the uncinate fasciculus and right SLF; negative correlation between FA and severity of ASD symptoms
Peterson *et al.* (2015) [101]	9–12 years	Global white matter	ROI; ANCOVA; LDDMM	↑ MD through left hemisphere, especially outer-zone of cortical WM
Kumar *et al.* (2010) [106]	2–9 years	Global white matter tracts	TBSS; DT; ANOVA; FSL	↓ FA in right uncinate fasciculus, right cingulum, and CC; ↑ MD in right arcuate fasciculus
Weinstein *et al.* (2011) [107]	2–4 years	Global white matter tracts	TBSS; DT; FSL	↑ FA in CC, left SLF and bilateral cingulum
Jou *et al.* (2011) [88]	7–15 years	Global white matter tracts	TBSS; FSL; SPSS	↓ FA in general association and projection tracts
Shukla *et al.* (2010) [85]	12–13 years	CC; internal capsule; middle cerebellar peduncle	TBSS; ROI; VBM; FSL; SPM	↓ FA ↑ MD for global white matter tracts
Walker *et al.* (2012) [108]	3–7 years	Global white matter tracts	TBSS; FSL	↓ FA in various areas; ↑ MD in posterior brain regions

WM, white matter; GM, grey matter; RD, radial diffusivity; FA, fractional anisotropy; MD, mean diffusivity; ROI, region of interest; TBSS, tract-based spatial statistics; DT, deterministic tractography; PT, probabilistic tractography; GLM, general linear models; ANOVA, analysis of variances; LDDMM, large deformation diffeomorphic metric mapping; VBM, voxel-based morphometry; SPM, FSL, DTIstudio, DTIprep, SPSS, and SAS are data analysis packages.

### Tractography

The majority of tractography studies have found reduced FA and increased MD, indicating more WM microstructural disorganization in the frontal and temporal lobes or dominant tracts. For example, Sundaram *et al.* (2008) applied deterministic tractography to analyze WM abnormalities in the frontal lobe and checked for short-range connectivity changes in ASD children. They reported higher MD in the whole frontal lobe as well as reduced FA for short-range fibers in ASD children [91]. Hong *et al.* (2011) also used deterministic tractography to examine connectivity in the CC in HFA children. They revealed decreased WM density in the anterior third of the CC, as well as higher MD and a lower fiber number in the anterior third of the transcallosal fiber tracts [92]. Using deterministic tractography, Nagae *et al.* (2012) revealed higher MD in the temporal portion of the left superior longitudinal fasciculus in ASD children with language impairment, as well as a significant negative correlation between MD and language score [93]. Wolff *et al.* (2012) investigated WM fiber tracts in a group of high-risk infants from 6 to 24 months old. Higher FA values were found in the fornix, uncinate fasciculus, and inferior longitudinal fasciculus at 6 months, whereas no such differences were observed at 12 months [94]. Nair *et al.* (2013) used both fMRI and probabilistic DTI tractography to assess the integrity of thalamo-cortical connectivity in children and adolescents with ASD. They reported increased MD in the thalamo-cortical connections, and decreased functional connectivity in the thalamo-cortical circuitry, as well as a negative correlation between the fronto-thalamic FA and the social and total Autism Diagnostic Observation Schedule score [95]. More recently, Joseph *et al.* (2014) applied both structural MRI and probabilistic DTI tractography to investigate the anatomo-behavioral relationship addressing language ability in young ASD children. In the ASD children, they found no difference in GM asymmetries but decreased leftward volume and radial diffusivity of the arcuate fasciculus [96].

### Voxel-Wise Analysis

Voxel-wise analysis is used to find areas of significant difference and to overcome possible user bias. Cheung *et al.* (2009) reported reduced FA in bilateral prefrontal and temporal regions as well as the frontal striato-temporal and posterior brain pathways that are associated with communication and social reciprocity impairment and repetitive behavior [97]. Ke *et al.* (2009) investigated the WM abnormalities in a group of Chinese HFA children. The voxel-wise whole-brain analysis of FA showed decreased WM density in the right frontal lobe, left parietal lobe, and right anterior cingulate [98]. Barnea-Goraly *et al.* (2010) found reduced FA in age-matched unaffected siblings as compared to children with ASD, suggesting that this may be a potential marker of genetic risk [99]. Poustka *et al.* (2012) revealed decreased FA values in the uncinate fasciculus and right superior longitudinal fasciculus as well as a negative correlation between the FA values of the affected fiber tracts and the severity of ASD symptoms [100]. A more recent study by Peterson *et al.* (2015) found widespread increases in MD in many regions of the left hemisphere in children with ASD as compared to typically-developing children [101], supporting the left hemispheric abnormality or atypical hemispheric dominance that has been hypothesized in ASD for the past three decades [102–104]. Peterson *et al.* (2015) used an atlas-based ROI analysis in HFA children and found significantly increased MD of the outer-zone cortical WM, suggesting hypomyelination and increased short-range cortico-cortical connections which might be due to the early WM overgrowth [101].

### Tract-Based Spatial Statistics (TBSS)

A further innovative method of DTI analysis is TBSS [105]. This method provides advantages over traditional voxel-wise analysis, since it does not require smoothing and allows for a higher spatial comparability. A mean FA skeleton is built and thresholded to exclude areas of high inter-individual variability. Each individual’s FA map is then projected onto the skeleton to collect standard voxel-wise FA statistics across all the individuals. Kumar *et al.* (2010) combined deterministic tractography and TBSS to investigate the CC region in 5-year-old ASD children. They found lower FA, higher MD, larger numbers of streamlines and voxels, and longer streamlines as well as correlation between macrostructural changes in the uncinate fasciculus and the score on the Gilliam Autism Rating Scale [106]. Weinstein *et al.* (2011) reported increased FA within the genu and body of the CC, left superior longitudinal fasciculus, and right and left cingulum in a group of very young ASD children using TBSS. The tractography revealed that the increased FA was concentrated in the mid-body of the CC and in the left cingulum [107]. Jou *et al.* (2011) found reduced FA in the forceps minor, inferior fronto-occipital fasciculus, and superior longitudinal fasciculus [88]. Shukla *et al.* (2011) demonstrated reduced FA and higher MD in the CC, anterior and posterior limbs of the internal capsule, inferior longitudinal fasciculus, inferior fronto-occipital fasciculus, superior longitudinal fasciculus, cingulum, anterior thalamic radiation, and corticospinal tract [85]. Similarly, Walker *et al.* (2012) revealed decreased FA values in various brain regions and increased MD in some tracts [108].

#### Conclusions

Studies using voxel-wise analysis have revealed decreased FA and increased MD in the major WM tracts of ASD children. However, the voxel-wise analysis of DTI has methodological disadvantages such as the dependency on the size of the smoothing kernel and the hindrance of confident conclusions [30]. TBSS overcomes these flaws and has been used in the majority of more recent and on-going tractography studies. Predominantly the latter have revealed reduced FA and higher diffusivity in the main tracts in ASD children, including the uncinate fasciculus, arcuate fasciculus, cingulum bundle, inferior longitudinal fasciculus, and inferior fronto-occipital fasciculus.

### Resting-State Functional Magnetic Resonance Imaging

Brain connectomics is a relatively new field of research that maps the brain’s large-scale functional networks “at rest”. Correlated and anti-correlated fMRI signals are measured without performing specific tasks. These signals imply a functional connectivity that reflects structural connectivity [109, 110]. They can be used to explore both the spatial and temporal deviations of the topology of atypical neurodevelopmental processes [111]. Table 3 summarizes the findings of rs-fMRI studies in children.

**Table 3:** Resting-state functional MRI studies in ASD children.

Reference	Age range (years)	Seed brain regions	Findings in ASD group
Di Martino *et al.* (2011) [114]	7–13	Striatal regions (caudate, putamen)	↑ Connectivity in striatal-cortical circuitry ↑ Striatal functional connectivity with the pons ↑ Connectivity of brainstem area, with bilateral insular regions
Lynch *et al.* (2013) [115]	7–12	Precuneus	↓ Connectivity to cuneus, caudate, and thalamic nuclei
		Posterior cingulate cortex	↑ Connectivity to medial and anterolateral temporal cortex, lingual gyrus, posterior parahippocampal gyrus, temporal pole, entorhinal cortex, and perirhinal cortex within the anterior aspect of the medial temporal lobe
		Retrosplenial cortex	↑ Connectivity to inferior frontal and middle frontal gyrus, dorsal medial prefrontal cortex, posterior insular cortex, lingual gyrus, posterior parahippocampal gyrus, temporal pole, posterior superior temporal sulcus, and anterior supramarginal gyrus
Uddin *et al.* (2013) [116]	7–12	Anterior cingulate cortex	↑ Connectivity to superior frontal gyrus, thalamus, and bilateral insular cortex
		Precuneus	↑ Connectivity to posterior cingulate cortex and left angular gyrus
		Superior temporal gyrus	↑ Connectivity to middle temporal gyrus
		Postcentral gyrus	↑ Connectivity to precentral gyrus, left posterior insular cortex, and thalamus
		Lateral occipital cortex	↑ Connectivity to intracalcarine cortex, and occipital pole
Wiggins *et al.* (2011) 119]	10–18	Right superior frontal gyrus	↓ Connectivity to posterior superior frontal gyrus
Rudie *et al.* (2012) [118]	10–15 (+/-MET mutation)	Posterior cingulate cortex	↓ Connectivity of overall default mode network ↓ Connectivity to medial prefrontal cortex in MET-homozygous ↓ Connectivity to medial prefrontal cortex within ASD group
Abrams *et al.* (2013) [117]	8–12	Posterior superior temporal sulcus	↓ Connectivity of bilateral ventral tegmental area, nucleus accumbens, putamen of basal ganglia, ventromedial prefrontal cortex, left caudate, anterior insular cortex, and orbitofrontal cortex
Nair *et al.* (2013) [95]	9–17	Right thalamus Thalamus	↑ Connectivity to temporal areas ↓ Connectivity to prefrontal, parietal-occipital, and somatosensory cortical regions

In an attempt to elucidate putative neurobiological underpinning mechanisms, Just *et al.* (2004) proposed the “hypoconnectivity theory of ASD”, claiming that the deficit of integration of neuronal information in ASD might be associated with an overall under-functioning of the brain’s integrative circuitry [112]. However, Courchesne and Pierce (2005) suggested that the developmental trajectory for functional brain connectivity in ASD individuals is characterized by both an early local hyperconnectivity and a long-distance hypoconnectivity of the prefrontal cortex based on the findings of an increased short-range (local) connectivity within the frontal lobe but a decreased degree of functional long-range (global) connectivity with the rest of the brain [113]. Di Martino *et al.* (2011) found only hyperconnectivity in the superior temporal gyrus, insula, and brainstem areas [114]. Moreover, Lynch *et al.* (2013) and Uddin *et al.* (2013) both demonstrated increased connectivity in the cingulate cortex and inferior and superior frontal gyri, as well as other specific areas [115, 116]. However, other childhood ASD studies mostly revealed reduced long-range connectivities across different brain regions [95, 117–119]. Thus, it has been suggested that the increased local connectivity in ASD children results from overcompensation for the reduced long-range connectivity [95].

### ^1^H-MRS Metabolite Spectrum

Neurochemicals are involved in cortical activity as well as in the metabolic processes in the brain. Each metabolite has a unique chemical shift which acts as a signature that is used for the quantification of that specific metabolite. The most common method of metabolite quantification *in vivo* is through the proton resonance of hydrogen (^1^H) atoms [120]. ^1^H-magnetic resonance spectroscopy (^1^H-MRS) is a non-invasive imaging technique that estimates specific chemical metabolites [121]. Previous studies mainly used ^1^H-MRS to quantify creatine and phosphocreatine (Cr+PCr), a measure of cellular energy metabolism [122]; N-acetylaspartate (NAA), a marker of neuronal density and activity [123]; choline-containing compounds, a measure primarily reflecting the constituents of cell membranes [122]; and glutamine/glutamate/gamma-aminobutyric acid (GABA) (“Glx”). Recent ^1^H-MRS studies in ASD patients showed generally decreased NAA [124, 125], Cr+PCr, choline, and Glx [125], as well as increased Glx in adults [126]. However, previous studies also exhibited some inconsistencies, such as decreased [127–131], unchanged, or increased NAA in ASD children compared with controls [127, 132–135]. In the following, we categorize the recent ^1^H-MRS studies in young individuals with ASD according to the four main detectable chemical metabolites: Cr+PCr, NAA, choline, and Glx. Table 4 summarizes the findings of MRS studies in children.

**Table 4:** MRS studies in ASD children.

Reference	Age range (years)	Cr+PCr	NAA	Cho	Glx	Findings in ASD group
Friedman *et al.* (2003) [129]	3–4	√	√	√		↓ Cr+PCr in frontal, parietal, temporal, occipital and thalamus ↓ NAA in bilateral frontal, parietal, and cingulate areas, and in right superior temporal gyrus and left putamen ↓ Cho in cortical areas, temporal lobes, and thalamus
DeVito *et al.* (2007) [128]	6–17	√	√	√	√	↓ Cr+PCr in GM of frontal, temporal, occipital cortices and cerebellum ↓ Cho in cortical areas, temporal lobes, and thalamus ↓ Glx in GM of frontal, occipital, and temporal regions as well as cerebellum
Hardan *et al.* (2008) [130]	8–15	√	√		√	↓ Cr+PCr in bilateral thalamus ↓ Cho in cortical areas, temporal lobes, and thalamus ↑ Glx of thalamus is associated with abnormal sensory sensitivity and deficits in body movement modulation
Corrigan *et al.* (2013) [138]	3–4	√	√	√	√	↓ Cr+PCr in GM and WM generally ↓ Cho in cortical areas, temporal lobes, and thalamus generally
Levitt *et al.* (2003) [131]	5–16	√	√	√		↓ Cr+PCr in frontal, parietal, temporal, occipital, and thalamus ↓ NAA in bilateral frontal and parietal areas, and in the left caudate ↓ Cho in cortical areas, temporal lobes, and thalamus ↑ Cho in caudate, anterior cingulate cortex and hippocampus-amygdala complex
Friedman *et al.* (2006) [144]	3–4	√	√	√	√	↓ NAA in both GM and WM generally ↓ Cho in cortical areas, temporal lobes, and thalamus
Corrigan *et al.* (2013) [138]	3–4	√	√	√	√	↓ NAA in both GM and WM in cortical regions ↓ Glx in both cortical GM and WM generally
Fujii *et al.* (2010) [145]	2–13	√	√	√		↓ NAA/Cr+PCr in anterior cingulate cortex and a deficit in executive functions
Fayed *et al.* (2005) [178]	2–10		√	√		↓ Cho in both GM and WM of cortical areas, temporal lobes and thalamus
Vasconcelos *et al.* (2008) [148]	6–10	√	√	√	√	↓ Cr+PCr in cerebellum and striatum ↓ NAA in frontal areas ↑ Cho in caudate, anterior cingulate cortex, and hippocampus-amygdala complex
Gabis *et al.* (2008) [146]	8–14		√	√		↓ NAA in Hippocampus-amygdala complex ↑ Cho in caudate, anterior cingulate cortex, and hippocampus-amygdala complex
Doyle-Thomas *et al.* (2014) [151]	7–18	√	√	√	√	↓ Glx/Cr+PCr in cerebellum and ↑ Glx/Cr+PCr in putamen ↓ NAA/Cr+PCr in thalamus and ↑ NAA/Cr+PCr in caudate

Cho, Choline; Cr+PCr, creatine and phosphocreatine; Glx, glutamine,glutamate and GABA; GM, grey matter; NAA, N-acetylaspartate; WM, white matter.

### Creatine and Phosphocreatine

In ^1^H-MRS, Cr and PCr are quantified together [136]. They play a crucial role in the adenosine triphosphate (ATP) and adenosine diphosphate (ADP) energy-transfer process. ATP results from oxidative phosphorylation in neuronal and glial mitochondria and glycolysis in the cytosol. A phosphate bond is released from ATP through the enzyme creatine kinase to store energy. The free phosphate bond then binds with Cr to form PCr. When cellular mitochondria require energy, ADP and a third phosphate bond are resynthesized to ATP via oxidative phosphorylation [122, 136, 137]. Cr is highly-expressed in the mitochondria of neurons and is crucial for cellular energy production and in the maintenance of cortical homeostasis [122, 137].

In children with ASD, reduced Cr+PCr levels have been reported across the cortical regions [128–130, 138]. Turner and Gant (2014) reviewed the biochemistry of Cr and revealed an association between reduced Cr+PCr and ASD-like behaviors, such as abnormal learning skills, intellectual disability, and repetitive and compulsive behaviors. This correlation could be due to delayed or impaired axon growth during brain development, in which Cr+PCr are crucial elements [139]. However, no correlation between Cr+PCr levels and autistic symptom severity has been found in children with ASD [140]. Moreover, cortical Cr+PCr is commonly considered to be stable and has been extensively used as a reference for other metabolites [120, 122, 127, 136].

### N-Acetylaspartate (NAA)

NAA is synthesized in the mitochondria of neurons and is catabolized in glial cells and oligodendrocytes, acting as a precursor of fatty-acids to form the myelin around axons [137]. Quantified by ^1^H-MRS, the NAA level is considered to reflect neuronal density, integrity, and metabolism [104, 131, 136, 141]. It is well accepted that NAA is comparable across both GM and WM, while it is more abundant in WM than in GM, due to its role in transmission of action potentials [142, 143].

Research on NAA has revealed a consistent phenomenon, i.e., it is generally reduced in children with ASD. In a group of 3–4 year-old ASD children, Friedman *et al.* (2003) reported NAA reduction in the frontal, parietal, and cingulate areas of both hemispheres, as well as in the right superior temporal gyrus and left putamen, compared with typically-developing children [129]. In an older group of ASD children with a mean age of 10.4 years, Levitt *et al.* (2003) revealed similar findings of reduced NAA in both left and right frontal and parietal areas, as well as in the left caudate. They also claimed that reduced parietal axon density, marked by reduced WM NAA, is associated with deficits in eye gaze, spatial perception, and memory [131]. Comparing children with ASD and those with pervasive developmental disorder, Friedman *et al.* (2006) found an extensively decreased NAA level in the ASD group across both GM and WM [144]. Similarly, Corrigan *et al.* (2013) found a general NAA reduction across most cortical regions in both GM and WM [138]. Interestingly, Fujii *et al.* (2010) reported a relationship between a reduced NAA/Cr+PCr ratio in the anterior cingulate cortex and a deficit in executive functions. Moreover, they also found a correlation between decreased NAA/Cr+PCr and social and communication disabilities [145].

### Choline

Choline is synthesized in the liver and is essential for the synthesis of the neurotransmitter acetylcholine. Choline-containing compounds along with membrane phospholipids make up 40% of myelin. In ^1^H-MRS, the choline level is highest in the WM [120]. The cortical choline levels indicate the equilibrium of neuronal cellular membrane phospholipid metabolism [120, 137, 146, 147].

In children with ASD, choline-containing compounds are generally decreased in cortical areas, the temporal lobes, and thalamus [128–131, 138], indicating a deficit in membrane phospholipid turnover. However, studies have also reported increased choline levels in ASD children in different brain regions, e.g. caudate, anterior cingulate cortex, and hippocampus-amygdala complex [131, 146, 148]. Even though the findings are contradictory, they revealed a similar phenomenon, that ASD children have neural maturation different from typically-developing children.

### Glx (Glutamate, Glutamine, and GABA)

Glx are the most abundant neurotransmitters (90% in synapses), taking up to 60%–80% of the glucose oxidation and energy consumption in cortical neurons [149, 150]. They are most concentrated in GM and play essential roles in neural migration, differentiation, and plasticity [125, 132, 136, 140]. In ASD children, De Vito *et al.* (2007) reported extensive reduction of Glx in the GM of frontal, occipital, and temporal regions as well as the cerebellum [128]. Corrigan *et al.* (2013) reported a general decrease in Glx in both cortical GM and WM [138]. Harden *et al.* (2008) pointed out that abnormal sensory sensitivity and deficits in body movement modulation are associated with increased thalamic Glx [130]. In addition, Doyle-Thomas *et al.* (2014) demonstrated that an increased thalamic Glx level is associated with disabled social interaction [151].

### MRI and Genetics

As outlined in the introduction, ASD has a strong genetic basis. Biological studies have revealed that many ASD-related genes influence the formation of neuronal circuits [21, 152, 153]. However, little is known about how these genes affect brain structure and function. As reviewed above, altered brain structures and functions have continuously been reported in ASD with a satisfying amount of consistency. Since brain structure and function are heritable [21, 154–156], gene-related imaging may serve to demonstrate the possible neural mechanisms through which phenotypic heterogeneity arises from genetic heterogeneity in ASD [21].

One innovative gene-related imaging study has compared ASD children carrying positively testable ASD-related gene(s) with typically-developing controls [118]. In particular, the authors were interested in the MET (Met Receptor Tyrosine) gene. They combined fMRI, rs-fMRI, and DTI in a large sample of ASD children (*n* = 164) with MET gene classification and controls. Indeed, the presence of risk alleles in ASD children had a significantly larger impact on functional connectivity than controls. In the ASD risk-allele group, the fMRI showed reduced functional connectivity from posterior cingulate to medial prefrontal cortex. DTI exhibited decreased FA in the splenium portion of the CC that connects to the posterior cingulate. Moreover, group-by-genotype interactions revealed more deactivation of the posterior cingulate, medial prefrontal, and primary auditory cortices, lower functional connectivity of these regions, and lower WM integrity in tracts connecting these regions (splenium, cingulum, and superior and inferior longitudinal fasciculus). Investigating the impact of another ASD-related gene (CNTNAP2; contactin-associated protein-like 2), Scott-Van Zeeland *et al.* (2010) revealed that risk-allele-carrying ASD children showed a pattern of diffusely increased functional connectivity between the frontal cortex and temporal lobes, while typically-developing controls exhibited a clear connectivity between frontal cortex- and language-related cortical regions [157].

According to these limited findings, we can only speculate that ASD-related genes may contribute to atypical connectivity and development. More studies combining genetic information and multimodal neuroimaging data are needed to understand the relationship between heterogeneous neuropathological phenotypes and the clinical manifestations of ASD, as well as to improve diagnostic tools and treatment strategies [158].

### Support Vector Machine Technologies

The findings of MRI studies have substantially advanced our understanding of the neural mechanisms underpinning ASDs. However, the integration of neuroimaging tools into clinical practice has so far been limited, partly because it is unclear which information revealed by these tools is relevant to diagnosis and treatment decisions. Support Vector Machine (SVM) technology is a specific type of supervised machine learning that aims to classify data points by maximizing the margin between classes in a high-dimensional space [159, 160]. The optimal SVM algorithm is developed through a “training” phase in which training data are used to develop an algorithm able to discriminate between groups previously defined by the operator (e.g. patients *versus* controls). Once a so-called “decision function” or “hyperplane” is learned from the training data, it can be used to predict the class of a new test example. The interesting aspect of the SVM is that it is multivariate and takes into account inter-regional correlations. Therefore, it is extremely well-suited to take into account and test subtle differences in intra-regional correlations of brain metabolism, function, and anatomy [161, 162]. This may be of particular relevance to ASD, in which the abnormalities are usually in the development of the whole neural system rather than isolated regions.

SVMs have been widely used to aid the diagnosis of neurological disorders, such as Alzheimer’s disease [163–166] and Parkinson’s disease [167, 168], as well as to identify those brain areas in stroke patients that are involved in a particular (cognitive) function [169, 170]. Among ASD patients, the SVM was first applied in adults. Singh *et al.* developed a diagnostic model generated by the LPboost-based algorithm to distinguish autistic children from controls, based on voxel-wise cortical thickness and ~40,000 points for each individual; they reported 89% classification accuracy based on cross-validation [171]. Ecker *et al.* (2010) applied SVM classifiers to investigate the predictive value of whole-brain structural volumetric changes in ASD, and obtained 81% classification accuracy based on cross-validation [172]. More recently, Ecker *et al.* (2013) pooled regional WM and GM volumes in ASD patients using SVMs and classified ASD patients with a high true positive rate [173]. As for ASD children, the number of reports is relatively limited. In a group of ASD children (mean age 9.2 ± 2.1 years), Jiao *et al.* (2010) used 4 different machine-learning techniques (including SVMs) to generate diagnostic models from both the VBM and SBM results. They reported a better classification performance for the thickness-based data than those based on regional volumes [44]. Lately, Jin *et al.* (2015) used an SVM classifier to identify high-risk ASD infants from WM tracts and whole-brain connectivity. Their proposed function achieved an accuracy of 76% and an area of 0.8 under the receiver operating characteristic curve [174].

To conclude, SVM technology is extremely promising in its contribution to an accurate and valid diagnosis of ASD. However, for ASD in children, the application of SVM is still at the beginning. More studies are needed to produce reliable diagnostic models based on imaging data.

### Summary and Future Direction

The main findings of MRI studies in children are summarized in Table 5.

**Table 5:** Summary of main MRI findings.

MRI approaches	Findings
Structural MRI	*Cortex*
	Increased total GM and WM volumes
	Increased GM and WM volumes in frontal and temporal areas
	Increased cingulate cortex
	Atypical variation of cortical thickness in frontal, temporal, and parietal lobes
	*Cerebellum and subcortical areas*
	*Cerebellum*
	Increased total volume
	Increased GM volume
	Decreased WM density
	*Amygdala*
	Increased volumes in younger children bilaterally
	Trajectory of development of amygdala follows overall trajectory of TBV
	*Corpus callosum*
	Decreased overall size
	Increased regional volume
	*Basal ganglia*
	Increased caudate volume
	Atypical shapes of the structures
	*Hippocampus*
	Increased size of hippocampi in young children
	Enlargement located especially on the right side
	No difference between sides in older children
Diffusion tensor imaging	Decreased FA in the whole brain, frontal lobe, arcuate fasciculus, across the entire CC, and in anterior thalamic radiation
	Increased FA in arcuate fasciculus and in CC in young children
	Increased MD in whole brain, frontal and temporal lobes, and across the entire CC
Resting-state fMRI	Altered functional connectivity in the default mode network
	Hyper-connectivity in striatal-cortical circuitry, precuneus, cingulate cortex, and temporal-frontal circuity
	Under-connectivity in anterior-posterior connections
Magnetic resonance spectroscopy	Decreased NAA levels in general GM and WM, especially in frontal, temporal, cingulate, and caudate areas
	Decreased Cr+PCr levels in general GM and WM, especially in frontal, parietal, temporal, occipital cortex, and thalamus
	Decreased choline levels in cortical areas, temporal lobes, and thalamus
	Increased choline levels in caudate, anterior cingulate cortex, and hippocampus-amygdala complex
	Decreased Glx in GM of frontal, occipital, temporal cortex, and cerebellar regions
	Increased Glx in thalamus and putamen

Young ASD children exhibit differences in brain morphometry, neurochemical components, and structural and functional connectivity. So far, a diverse set of potential biomarkers including genetic, biochemical, morphological, hormonal, immunological, neuropathological, neuropsychological, and behavioral biomarkers have been identified. However, for most of these markers it is not yet clear if they are contributing factors to the development of ASD or are a result of another underlying abnormality. Longitudinal studies − especially covering very young children with ASD − may help answer this question.

It should be kept in mind that MRI techniques are limited to a certain level of spatial and temporal resolution. If this level is not precise enough to visualize the synaptic or neuronal abnormalities where the core features or heterogeneity of ASD arises neuroimaging may ultimately not be the best tool for parsing these differences. However, in combination with post-mortem tissue analysis or animal models, neuroimaging studies have the potential to provide a critical intermediate step between the genetic basis and the phenotype. Such approaches allow us to deconstruct the conceptions of ASD deeply to where they can be grounded in biology [87, 175].

While most of the studies reviewed here are still characterized by methodological variability and relatively small sample sizes, studies with pooled data samples and homogeneous analytical approaches are needed. Large-scale collaboration networks of that type are ABIDE (“Autism Brain Imaging Data Exchange”) [114, 176, 177] and NDAR (“National Database for Autism Research”). They combine imaging data from multiple sites and integrate both genetic and behavioral data. These projects will allow researchers to achieve sufficient power to detect true brain-gene-behavior relationships [21]. In general, multi-site and multi-modal studies based on large patient groups is one of the key strategies to increase the probability of the discovery of new effective biomarkers in ASD and to comprehensively characterize the clinical, behavioral, and cognitive symptoms of this disorder.

Along with the growing numbers of gene-related MRI studies, the application of multimodal MRI scanning and collaboration networks, as well as advanced analytical methods are needed. Techniques, such as a machine learning classifier or prediction, may serve to identify patterns of biomarkers across different modalities, relevant to clinical diagnosis and genotype, severity rating, prognosis, and/or response to treatment. Last but not least, further large-sample studies on ASD children are needed to search for reliable biomarker patterns and diagnostic models based on imaging data.
